# Risk of hip fracture among older people using anxiolytic and hypnotic drugs: a nationwide prospective cohort study

**DOI:** 10.1007/s00228-014-1684-z

**Published:** 2014-05-09

**Authors:** Marit Stordal Bakken, Anders Engeland, Lars B. Engesæter, Anette Hylen Ranhoff, Steinar Hunskaar, Sabine Ruths

**Affiliations:** 1 Department of Global Public Health and Primary Care, University of Bergen, PB 7804, 5020 Bergen, Norway; 2 Kavli Research Centre for Geriatrics and Dementia, Haraldsplass Deaconess Hospital, PB 6165, 5892 Bergen, Norway; 3 Division of Epidemiology, Department of Pharmacoepidemiology, Norwegian Institute of Public Health, Kalfarveien 31, 5018 Bergen, Norway; 4 Norwegian Arthroplasty Registry, Department of Orthopaedics and Department of Clinical Medicine, Haukeland University Hospital and University of Bergen, Jonas Lies veg 87, 5021 Bergen, Norway; 5 Department of Clinical Sciences, University of Bergen, PB 1400, 5021 Bergen, Norway; 6 National Centre for Emergency Primary Health Care, Uni Research Health, Kalfarveien 31, 5018 Bergen, Norway; 7 Research Unit for General Practice, Uni Research Health, Kalfarveien 31, 5018 Bergen, Norway

**Keywords:** Anxiolytics, Hypnotics, Hip fracture, Time of fracture, Pharmacoepidemiology, Population-based registry

## Abstract

**Purpose:**

Anxiolytics and hypnotics are widely used and may cause injurious falls. We aimed to examine associations between exposure to anxiolytics and hypnotics and the risk of hip fracture among all older people in Norway. Further, we wanted to examine associations between exposure to hypnotics and time of fracture.

**Methods:**

A nationwide prospective cohort study of people in Norway born before 1945 (*n* = 906,422) was conducted. We obtained information on all prescriptions of anxiolytics and hypnotics dispensed in 2004–2010 (the Norwegian Prescription Database) and all primary hip fractures in 2005–2010 (the Norwegian Hip Fracture Registry). We compared the incidence rates of hip fracture during drug exposure and non-exposure by calculating the standardized incidence ratio (SIR).

**Results:**

Altogether, 39,938 people (4.4 %) experienced a primary hip fracture. The risk of hip fracture was increased for people exposed to anxiolytics (SIR 1.4, 95 % confidence interval (CI) 1.4–1.5) and hypnotics (SIR 1.2, 95 % CI 1.1–1.2); the excess risk was highest regarding short-acting benzodiazepine anxiolytics (SIR 1.5, 95 % CI 1.4–1.6). Benzodiazepine-like hypnotics (z-hypnotics) were associated with higher excess risk of hip fracture at night (SIR 1.3, 95 % CI 1.2–1.4) than during the day (SIR 1.1, 95 % CI 1.1–1.2).

**Conclusions:**

Older people had an increased risk of hip fracture during anxiolytic or hypnotic drug use, including short-acting benzodiazepine anxiolytics and z-hypnotics that were previously considered less harmful; cautious prescribing is therefore needed. People using z-hypnotics were at greatest excess risk at night; this association deserves further investigation.

## Introduction

Hip fractures are highly prevalent among older people, with great implications for morbidity and mortality [1, 2]. Thus, identifying and reducing modifiable risk factors are essential. Anxiolytics (drugs that reduce anxiety, mainly benzodiazepines) and hypnotics (drugs that reduce sleep disturbances, mainly benzodiazepine-like drugs, z-hypnotics: zolpidem, zopiclone and zaleplon) independently increase the risk of falling because of sedation, impaired balance and impaired cognition [3–5]. Benzodiazepines have been associated with increased risk of hip fracture, but estimates diverge [6–10]. Z-hypnotics have previously been considered less harmful [11], but growing evidence suggests that they are not safer than benzodiazepines regarding either falls [12] or hip fractures [13, 14].

Use of anxiolytic and hypnotic drugs increases with age, and these drugs are commonly used by people most prone to adverse drug effects and hip fractures [15, 16]. Long-term use is widespread [16] although advised against [17]. In Norway, 15 % of the people aged 70 years or older receive at least one anxiolytic drug prescription yearly [18]; overall, European numbers range from 10 % (community dwellers) to 55 % (nursing home patients) [19–23]. Hypnotics (mostly z-hypnotics) are prescribed to 26 % of the people in Norway 70 years or older and to 52 % of those older than 90 years [18], in accordance with studies in Europe [15, 21] and the USA [24]. Knowledge is scarce regarding associations between the use of hypnotics and the time of hip fracture.

We conducted a prospective cohort study to examine associations between exposure to anxiolytics and hypnotics and the risk of hip fracture among the whole population of Norway aged 60 years and older in 2005–2010. If we found associations, we would aim to estimate the attributable risk of hip fracture. Further, we wanted to examine associations between exposure to hypnotics and the time of hip fracture.

## Methods

### Design

We performed a nationwide prospective cohort study based on merged data from the Norwegian Prescription Database (NorPD) [25], the Norwegian Hip Fracture Registry [26] and the Central Population Registry [27]. The study period lasted from January 1, 2005, to December 31, 2010. The data sources and methods have previously been described in detail [28, 29].

### Data sources

The NorPD was established in January 2004 [25]. It contains information on all prescription drugs purchased by individual patients at all pharmacies in Norway. The NorPD does not contain indications for prescriptions or individual information on medication dispensed to people living in nursing homes (about 40,000 at any time—4.4 % of the study population). The following data were extracted: all prescriptions of anxiolytics and hypnotics dispensed from January 1, 2004 (prescriptions from 2004 necessary to be able to identify the users when the study started), until December 31, 2010, to people born before 1945 by the items’ generic name, Anatomical Therapeutic Chemical (ATC) system code [30] and defined daily dose (DDD) [30]. In Norway, all anxiolytics and hypnotics are prescription drugs only.

The Norwegian Hip Fracture Registry was established in January 2005 [26]. This national registry contains information about fractures and surgery performed on people for hip fracture at all 55 hospitals in Norway performing such surgery [31]. We extracted data on the time and date of primary hip fracture (date of surgery in case of missing information) for the period January 1, 2005, until December 31, 2010. The Norwegian Hip Fracture Registry does not contain information on the place where fractures occurred, such as a nursing home.

The Central Population Registry contains demographic information on the entire population of Norway [27]. We extracted data on birth year, sex and date of death or emigration if applicable. All residents of Norway after 1960 have been assigned a unique 11-digit personal identity number, which we used to link the selected variables from the various registries.

### Study population

The study population was all residents of Norway born before 1945 and living in Norway on January 1, 2005. We followed them until the day of any first hip fracture, emigration or death or until the end of the study period.

### Medications studied

We included the following medications in this study:

- ATC code N05B, anxiolytics, main indication: anxiety
  - N05BA, benzodiazepine derivates (diazepam, oxazepam, alprazolam)
  - N05BB, other anxiolytics (hydroxyzine)
- ATC code N05C, hypnotics, main indication: sleep disturbances
  - N05CD, benzodiazepine derivates (nitrazepam, flunitrazepam, midazolam)
  - N05CF, benzodiazepine-related drugs (zopiclone, zolpidem)
  - N05CH, melatonin receptor agonists (melatonin)

We excluded the following drugs because of indications other than anxiety and sleep disturbances or very rare use (clomethiazole, chlordiazepoxide, lorazepam, bromazepam, clobazam, meoprobamate, busperione, barbital, flurazepam, triazolam, zaleplon and scopalamine). We also classified the benzodiazepine anxiolytics and hypnotics according to their half-lives (Table 2, footnote).

### Exposure

The DDD is the assumed average maintenance dose per day for a drug used for its main indication in adults [30]. Prescribed daily dose (PDD) and actual drug consumption vary within a population. The NorPD does not include information on PDD or on whether or when the purchasers consumed the dispensed drugs; we needed to make assumptions about drug exposure. We calculated the risk of hip fracture for various assumed total exposure times (3, 7 and 14 days and the number of days corresponding to the number of DDDs prescribed, respectively; we performed calculations for both 0.5 and 1.0 DDD). We assumed that people started using the drugs on the day they were purchased and that hypnotics were taken at bedtime.

We investigated both overall and recently started use of anxiolytic and hypnotic drugs. We defined overall use as any exposure to anxiolytics or hypnotics within the study period, including all exposure periods. We defined recently started use as the first 14 days of exposure to the drug in question after a 360-day washout period.

### Statistical analysis

We compared the incidence of hip fracture during the person-days exposed and unexposed to anxiolytics and hypnotics during the study period, by calculating standardized incidence ratios (SIRs) [29]. If a person received an anxiolytic or hypnotic prescription more than once during the study period, all exposed person-time periods were included in the calculations. An SIR greater than 1 indicates an increased risk of hip fracture associated with drug exposure. We adjusted the SIRs for sex, birth year and time period (divided into 2-month intervals).

We performed subanalyses for recently started drug use. Further, we conducted subanalyses for use of hypnotics and time of fracture, at night (20:00–7:59) or during the day (8:00–19:59).

We calculated the attributable risk of exposure to anxiolytics or hypnotics on hip fracture by dividing the observed minus the expected number of fractures during the number of person-days exposed to the drugs in question by the observed number of fractures in the study population.

## Results

The study population comprised 906,422 people with a mean age of 72.8 years (standard deviation (SD) 8.9 years) on January 1, 2005 (56 % women), and mean follow-up 5.2 years (SD 1.6). Figure 1 shows an overview of the data sources used and the data extracted. Altogether, 218,775 people died (53 % women) and 4,949 emigrated (44 % women).

**1:**
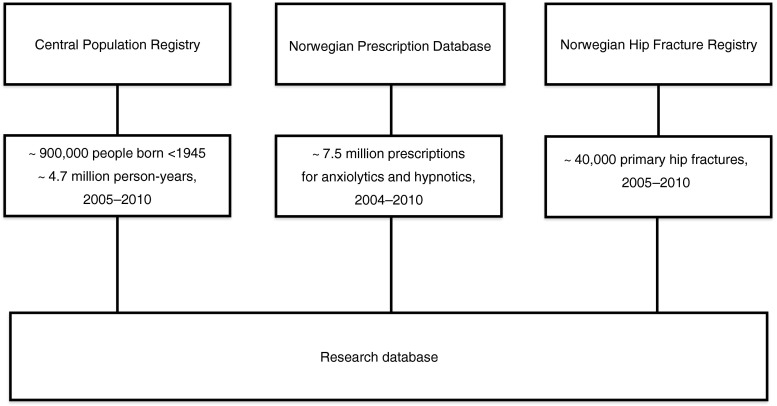
Data sources (nationwide registries) and data extracted for the research database. Prescriptions from 2004 needed to be able to identify users when the study started (January 1, 2005)

A total of 204,532 (23 %) people received at least one prescription for an anxiolytic during the study period; 69 % were women. Hypnotics were prescribed for 275,372 (30 %) people; 67 % were women. Z-hypnotics were the drugs most frequently used (Table 1). More women than men purchased all drug groups investigated; for both sexes, drug use was most prevalent among individuals born in 1925–1929 and 1930–1934.

**Table 1:** Percentage of people in Norway born before 1945 exposed to any anxiolytic or hypnotic drug and various therapeutic subgroups during 2005–2010. Individuals may have purchased more than one anxiolytic or hypnotic drug

	Anxiolytic and hypnotic drugs (percentage of people exposed at least once)						
	Anxiolytics			Hypnotics			
	Total	Benzodiazepine derivates	Other anxiolytics	Total	Benzodiazepine derivates	Benzodiazepine-related drugs Z-hypnotics	Melatonin receptor agonists
Total cohort (*n* = 906,422)	22.6	21.0	2.9	30.4	5.2	28.1	1.9
Women (*n* = 506,568)	27.8	26.2	3.3	36.4	6.3	33.6	2.4
Men (*n* = 399,854)	15.9	14.3	2.5	22.8	3.7	21.1	1.3

*Anxiolytics*, ATC code N05B: benzodiazepine derivates (diazepam, oxazepam, alprazolam) and other anxiolytics (hydroxyzine); *Hypnotics*, ATC code N05C: benzodiazepine derivates (nitrazepam, flunitrazepam, midazolam), benzodiazepine-related drugs, z-hypnotics (zopiclone, zolpidem) and melatonin receptor agonists (melatonin)

Altogether, 39,938 individuals (mean age 83.0 years; 72 % women) experienced a primary hip fracture during the study period. A total of 2,009 fractures (82 % among women) occurred during exposure to anxiolytics—35 % among those born in 1915–1924 and 41 % among those born in 1925–1934. A total of 6,583 fractures (80 % in women) occurred during exposure to hypnotics—47 % among those born in 1915–1924 and 36 % among those born in 1925–1934. In our study, 675 people (9 %) were exposed to both anxiolytics and hypnotics at the time of fracture. The prevalence of hip fracture was higher among exposed women than among exposed men within all birth cohorts.

The SIRs increased with increasing numbers of assumed exposed person-days from 3 to 14 days and remained largely stable when SIR was calculated for the number of days corresponding with 0.5 and 1.0 DDD (not shown). Because we found similar SIRs for 0.5 and 1.0 DDD and wanted to avoid misclassifying non-users as users, we considered the number of DDDs the best proxy for drug exposure.

The excess risk of hip fracture was more pronounced among exposed men than among exposed women. For anxiolytics, the SIR was 1.6 (95 % confidence interval (CI) 1.4–1.7) among men and 1.4 (95 % CI 1.4–1.5) among women and, for hypnotics, 1.3 (95 % CI 1.2–1.3) and 1.1 (95 % CI 1.1–1.2), respectively. Within all drug groups investigated, sex differences were most prominent in the youngest cohort born in 1935–1944 (not shown) and the excess risk of hip fracture among users decreased with increasing age (Table 2).

**Table 2:** Comparison of number of hip fractures (*n*) during exposed and unexposed person-time (standardized incidence ratio (SIR), 95 % CI) in the population of Norway born before 1945 and exposed to various anxiolytic and hypnotic drugs in 2005–2010, by sex and birth cohort (exposed person-days, DDD)

	Anxiolytics						Hypnotics				Combination	
	Anxiolytics (total)		Short-acting benzodiazepines		Long-acting benzodiazepines		Hypnotics (total)		Z-hypnotics		Any benzodiazepine or benzodiazepine-like drug	
	*n*	SIR	*n*	SIR	*n*	SIR	*n*	SIR	*n*	SIR	*n*	SIR
Total cohort	2,009	1.4 (1.4–1.5)	896	1.5 (1.4–1.6)	2,141	1.2 (1.2–1.3)	6,583	1.2 (1.1–1.2)	5,418	1.2 (1.1–1.2)	7,814	1.2 (1.2–1.2)
By sex												
Women	1,642	1.4 (1.4–1.5)	739	1.4 (1.3–1.5)	1,743	1.2 (1.2–1.3)	5,274	1.1 (1.1–1.2)	4,326	1.1 (1.1–1.2)	6,273	1.2 (1.1–1.2)
Men	367	1.6 (1.4–1.7)	157	1.7 (1.5–2.0)	398	1.3 (1.2–1.5)	1,309	1.3 (1.2–1.3)	1,092	1.3 (1.2–1.4)	1,541	1.3 (1.2–1.4)
By birth cohort												
1935–1944	410	2.7 (2.4–3.0)	148	2.4 (2.1–2.8)	349	2.6 (2.3–2.9)	787	2.0 (1.8–2.1)	676	1.9 (1.8–2.1)	1,020	2.1 (2.0–2.3)
1925–1934	819	1.6 (1.5–1.7)	343	1.6 (1.4–1.7)	774	1.4 (1.3–1.5)	2,341	1.3 (1.2–1.3)	1,986	1.3 (1.2–1.3)	2,844	1.3 (1.3–1.4)
1915–1924	712	1.1 (1.0–1.2)	369	1.2 (1.1–1.3)	890	1.0 (0.9–1.0)	3,098	1.0 (1.0–1.0)	2,501	1.0 (1.0–1.0)	3,552	1.0 (1.0–1.0)
<1915	68	1.1 (0.9–1.4)	36	1.2 (0.9–1.7)	128	1.1 (0.9–1.3)	357	1.0 (0.9–1.1)	255	1.0 (0.9–1.1)	398	1.0 (0.9–1.1)
Attributable risk		1.5		0.7		1.0		2.3		1.9		3.2

*Anxiolytics*, ATC code N05B: benzodiazepine derivates (diazepam, oxazepam, alprazolam) and other anxiolytics (hydroxyzine); *Hypnotics*, ATC code N05C: benzodiazepine derivates (nitrazepam, flunitrazepam, midazolam), benzodiazepine-related drugs or z-hypnotics (zopiclone, zolpidem) and melatonin receptor agonists (melatonin); *Short-acting benzodiazepines*, half-life <24 h: oxazepam, alprazolam and midazolam; *Long-acting benzodiazepines*, half-life >24 h: diazepam, nitrazepam and flunitrazepam; *Any benzodiazepine or benzodiazepine-like drug*: short-acting benzodiazepines + long-acting benzodiazepines + z-hypnotics (zopiclone, zolpidem)

The risk of hip fracture was elevated for people exposed to any anxiolytic (SIR 1.4, 95 % CI 1.4–1.5); the excess risk of hip fracture was higher among people exposed to short-acting benzodiazepines (SIR 1.5, 95 % CI 1.4–1.6) than among people exposed to long-acting benzodiazepines (SIR 1.2, 95 % CI 1.2–1.3). Further, the risk of hip fracture was elevated among people exposed to any hypnotic (SIR 1.2, 95 % CI 1.1–1.2) or z-hypnotics (SIR 1.2, 95 % CI 1.1–1.2), respectively.

Subanalyses for recently started drug use revealed excess risk of hip fracture for short-acting benzodiazepines (SIR 1.3, 95 % CI 1.0–1.7) and z-hypnotics (SIR 1.2, 95 % CI 1.0–1.5) only (not shown in tables). The time of hip fracture was available for 3,323 (51 %) of the people who experienced a hip fracture during exposure to hypnotics. Z-hypnotics were associated with higher excess risk of hip fracture at night (SIR 1.3, 95 % CI 1.2–1.4) than during the day (SIR 1.1, 95 % CI 1.1–1.2) (Table 3).

**Table 3:** Comparison of daytime (08:00–19:59) and nighttime (20:00–07:59) observed number of hip fractures (*n*) and excess risk of hip fracture (standardized incidence ratio (SIR), 95 % CI) in the population of Norway born before 1945 exposed to hypnotics in 2005–2010 (exposure 7 days, 14 days and DDD)

	Hypnotics (total)		Hypnotics day^a^		Hypnotics night^a^		Z-hypnotics day^a^		Z-hypnotics night^a^	
	*n*	SIR	*n*	SIR	*n*	SIR	*n*	SIR	*n*	SIR
Exposed person-days										
7	1,050	1.3 (1.2–1.5)	346	1.2 (1.1–1.3)	172	1.4 (1.2–1.6)	294	1.2 (1.1–1.4)	142	1.4 (1.2–1.6)
14	2,071	1.3 (1.2–1.3)	678	1.2 (1.1–1.3)	340	1.4 (1.3–1.6)	574	1.2 (1.1–1.4)	277	1.4 (1.2–1.5)
DDD	6,583	1.2 (1.1–1.2)	2,245	1.1 (1.1–1.2)	1,078	1.3 (1.2–1.4)	1,835	1.1 (1.1–1.2)	884	1.3 (1.2–1.4)
Attributable risk		2.3		2.1		4.0		1.7		3.3

*Hypnotics*, ATC code N05C: benzodiazepine derivates (nitrazepam, flunitrazepam, midazolam), benzodiazepine-related drugs or z-hypnotics (zopiclone, zolpidem) and melatonin receptor agonists (melatonin); *Z-hypnotics*, ATC code N05CF: benzodiazepine-related drugs (zopiclone, zolpidem)

^a^Time of fracture known in 51 % of cases (hip fractures occurring during exposure to hypnotic drugs)

### Attributable risk

The share of hip fractures attributable to exposure to anxiolytics was estimated at 1.5 % (short-acting benzodiazepines 0.7 % and long-acting benzodiazepines 1.0 %) and exposure to hypnotics at 2.3 % (z-hypnotics 1.9 %). The attributable risk of hypnotics was twice as high at night (4.0 %) as during the day (2.1 %). The corresponding figures regarding z-hypnotics were 3.3 and 1.7 %, respectively. When all benzodiazepines and benzodiazepine-like anxiolytics and hypnotics were grouped together, the attributable risk was estimated at 3.2 %.

## Discussion

We found increased risk of hip fracture in people exposed to anxiolytics and hypnotics, especially short-acting benzodiazepines. The excess risk of hip fracture associated with hypnotics was higher at night than during the day. About 3 % of all hip fractures were attributable to the use of anxiolytics or hypnotics.

### Benzodiazepines

Our results showing increased risk of hip fracture among users of benzodiazepine anxiolytics and hypnotics are in accordance with previous studies [6–9, 32]. This association probably results from increased risk of falling caused by side effects such as sedation, impaired balance and reduced cognition [9, 14, 33]. To our knowledge, no evidence indicates that benzodiazepines or hypnotics increase the risk of osteoporosis, in contrast to other psychotropic drugs such as antidepressants and antipsychotics [34].

Whether short-acting benzodiazepines or long-acting benzodiazepines pose the greatest risk of hip fracture is unclear [9]. Heterogeneity in study populations, study designs and drug groups investigated hampers comparisons [35]. A recent literature review including studies in Europe and the USA [9] revealed pooled relative risks of hip fracture of 1.23 for short-acting benzodiazepine and z-hypnotic users and 1.32 for long-acting benzodiazepine users. On the other hand, attributable risks were highest regarding short-acting benzodiazepines and z-hypnotics, since these were most widely used [9]. In a recent meta-analysis, the use of benzodiazepines, especially short-acting benzodiazepines, was associated with a statistically and clinically significantly increased risk of any fracture; the relative risk of hip fracture was 1.35 among users of any benzodiazepines [10].

We analysed short-acting benzodiazepines, long-acting benzodiazepines and z-hypnotics separately and found that the excess risk of hip fracture was higher among people using short-acting benzodiazepines than among those using long-acting benzodiazepines or z-hypnotics.

In accordance with previous studies, we found an increased risk of hip fracture associated with recently started use of short-acting benzodiazepines [36]. Growing evidence indicates a dose–response curve for fracture risk, starting already at very low (0.2 DDD) drug dosages [6, 37, 38]. Guidelines recommend non-pharmaceutical treatment options in anxiety of mild to moderate severity and insomnia and low dosages and short duration only when prescribing [17]. Still, benzodiazepines and z-hypnotics are commonly prescribed for older people at higher dosages and for longer periods [16].

### Z-hypnotics

The elevated risk of hip fracture identified among people using z-hypnotics is in accordance with previous studies [14, 39]. Z-hypnotics have previously been considered less harmful [11], and there has been an intentional shift from benzodiazepines to z-hypnotics. Nevertheless, observational studies suggest that z-hypnotics are not safer regarding falls [12] or hip fractures [13, 14], probably by inducing or worsening impairment of balance and cognition. Further, the effectiveness of z-hypnotics is limited among people aged 60 years or older, which has led to a great controversy on the use of these drugs among older people [24]. Cognitive behavioural therapy (including, e.g. stimulus control and sleep restriction), on the other hand, is highly effective [40].

We found an increased risk of hip fracture during the first 14 days among previous non-users, in agreement with Berry et al. [39], suggesting that short-term use may also be harmful. Our findings are clinically relevant because z-hypnotics are widely prescribed to (very) old people [41]. Their significance is further underlined by z-hypnotics being associated with the greatest overall impact on attributable risk estimates.

### Time of fracture

One may hypothesize that hypnotics protect against falls and hip fracture at night because the people taking these drugs would sleep and not stand up and use the bathroom. Nevertheless, z-hypnotics may cause balance impairment and confusion on awakening and thus increased risk of falls and hip fracture [12]. A previous study investigating diurnal fracture patterns has shown a daytime peak [42]. Among people with or without dementia using psychotropic drugs, this diurnal pattern was lacking, probably because of increased fracture risk at night [42]. In accordance with that study, we found higher excess risk and higher attributable risk of hip fracture associated with z-hypnotics at night than during the day.

### Age

Generally, we found the excess risk of hip fracture to be most evident within the youngest cohorts. We probably underestimated the risk of hip fracture among the oldest old because of two methodological issues discussed below: the lack of clinical information (confounding factors could not be adjusted for) and the systematic misclassification of the highly exposed [27] nursing home patients as drug non-users.

### Methodological considerations

The national health registries provided a unique opportunity to link complete data on anxiolytics and hypnotics purchased by an unselected community-dwelling older population with all primary hip fractures registered in Norway. The 6-year follow-up period, with all exposure periods included, revealed high numbers of cases, and the nationwide prospective study design prevented selection and information bias. Randomized controlled trials comparing short-acting benzodiazepines and long-acting benzodiazepines are not likely to be performed for ethical reasons.

The databases used have some limitations. The NorPD lacks individual information on medication dispensed to people living in nursing homes, leading to systematic misclassification of about 40,000 people at any time as drug non-users. Because nursing home residents have a high prevalence of both hip fracture and anxiolytic and hypnotic drug use [43], the excess risk of hip fracture has been underestimated among exposed people. The Norwegian Hip Fracture Registry comprises more than 80 % of all hip fracture operations in Norway [26], being somewhat less complete during the first years. The time of fracture was available in half the cases. There is no reason to suspect systematically biased underreporting; we found that about two thirds of the eligible cases occurred during daytime, in accordance with a recent study in Sweden [42].

A study in Sweden revealed the PDDs to be 0.42 for anxiolytics and 0.64 for hypnotics, respectively [44]. Our calculations based on drug exposure corresponding with 1.0 DDD may therefore have revealed conservative risk estimates. However, we wanted to avoid misclassifying non-users as users.

The Norwegian Hip Fracture Registry lacks information on other clinical conditions. Many factors may influence the risk of falls and fractures, such as acute and chronic somatic and mental health conditions, sleep disturbances and physical activity at night, balance impairment, frailty, lifestyle and concomitant drug use [45]. Nevertheless, previous studies have shown the excess risk of hip fracture associated with anxiolytics and hypnotics to remain when adjusting for cognitive and functional status, BMI and smoking [46], and concomitant drug use [8]. Thus, we chose not to adjust for concomitant drug use, which would also have introduced further uncertainty (due to the lack of clinical information).

There is no reason to suspect that the findings in this nationwide study should not be generalisable to other countries.

## Conclusion

Our nationwide, prospective study adds important knowledge on the excess risk of hip fracture for users of short-acting benzodiazepines and z-hypnotics, which were previously considered less harmful for use among older people. Since hip fractures are highly prevalent in this population, even a minor excess risk may cause great numbers of hip fractures, with major clinical and economic consequences.

Our results emphasize the need for careful consideration in treating old people with anxiety or insomnia. Growing evidence implies no reason to prefer short-acting benzodiazepines to long-acting benzodiazepine anxiolytics or z-hypnotics to benzodiazepine hypnotics with regard to the risk of hip fracture because even short-term use of these drugs is associated with increased risk. Thus, non-pharmaceutical treatment options should be given priority and efforts should be made to reduce dosages and withdraw drugs from long-term users. We found people using z-hypnotics to be at greatest risk at night; this association deserves further investigation.
